# A multi-million image Serial Femtosecond Crystallography dataset collected at the European XFEL

**DOI:** 10.1038/s41597-022-01266-w

**Published:** 2022-04-12

**Authors:** Henry J. Kirkwood, Raphael de Wijn, Grant Mills, Romain Letrun, Marco Kloos, Mohammad Vakili, Mikhail Karnevskiy, Karim Ahmed, Richard J. Bean, Johan Bielecki, Fabio Dall’Antonia, Yoonhee Kim, Chan Kim, Jayanath Koliyadu, Adam Round, Tokushi Sato, Marcin Sikorski, Patrik Vagovič, Jolanta Sztuk-Dambietz, Adrian P. Mancuso

**Affiliations:** 1 European XFEL, Holzkoppel 4, 22869 Schenefeld, Germany; 2 School of Chemical and Physical Sciences, Keele University, Staffordshire, ST5 5AZ United Kingdom; 3 Department of Chemistry and Physics, La Trobe Institute for Molecular Science, La Trobe University, Melbourne, 3086 Australia

**Keywords:** Scientific data, Proteins, X-ray crystallography

## Abstract

Serial femtosecond crystallography is a rapidly developing method for determining the structure of biomolecules for samples which have proven challenging with conventional X-ray crystallography, such as for membrane proteins and microcrystals, or for time-resolved studies. The European XFEL, the first high repetition rate hard X-ray free electron laser, provides the ability to record diffraction data at more than an order of magnitude faster than previously achievable, putting increased demand on sample delivery and data processing. This work describes a publicly available serial femtosecond crystallography dataset collected at the SPB/SFX instrument at the European XFEL. This dataset contains information suitable for algorithmic development for detector calibration, image classification and structure determination, as well as testing and training for future users of the European XFEL and other XFELs.

## Abstract (summary)

**Table:** 

Measurement(s)	lysozyme measurement
Technology Type(s)	X-ray crystallography

## Background & Summary

Serial femtosecond crystallography (SFX) utilises the ultrafast and ultrabright pulses of an X-ray free electron laser (XFEL) to overcome some of the challenges faced in conventional X-ray crystallography for biological structure determination^1^. Firstly, the ultrabright pulses provide the ability to measure sufficient X-ray diffraction from micrometer and sub-micrometer sized protein crystals^2^. Secondly, the brightness combined with the ultrafast X-ray pulse duration enables the collection of essentially radiation damage free^3^ diffraction data at room temperature^2^. The SFX method further enables structure determination in time-resolved systems where femtosecond time resolution is needed, such as in pump-probe^4–6^, irreversible or mixing experiments^7,8^. Hence SFX has significant potential as a tool for determining the structure of these challenging classes of biological molecules^9^.

The European XFEL (EuXFEL)^10^ is the first high repetition rate XFEL and uses a unique burst mode pulse structure to deliver up to 27000 electron bunches per second which are shared between the different self-amplified spontaneous emission (SASE) undulators^11^. The SPB/SFX instrument^12^ is located behind the SASE1 undulator and is capable of recording 3520 X-ray pulses per second with the MHz-capable, Adaptive Gain Integrating Pixel Detector (AGIPD)^13^. Bursts of X-ray pulses arrive at the instrument in trains of up to 352 pulses, with an intratrain repetition rate of up to 4.5 MHz and an intertrain rate of 10 Hz (enabling diffraction to be recorded at megahertz repetition rates).

The experimental challenges of increased repetition rate lie particularly in sample delivery and data analysis. SFX relies on illuminating a fresh crystal with each X-ray pulse, hence places a high demand on rapid and consistent sample delivery–typically in a liquid jet^14^.There is also an open question around the effects of XFEL induced shockwaves on crystals delivered in a liquid jet^15,16^. Generating 3520 diffraction images per second (~16 GB s^−1^) also places significant demand on data analysis. Each measured image needs calibration and classification followed by the extraction of crystallographic information, which requires a complex work flow. In SFX experiments, typically less than 10% of frames contain crystal diffraction, hence fast and accurate classification is critical for optimising sample preparation, sample delivery and efficient instrument operation.

This paper describes the deposition of an EuXFEL SFX dataset containing 19 million images^17^, recorded in approximately 1.5 hours by AGIPD, for structure determination of hen egg-white lysozyme (HEWL). HEWL has a well known structure, is very easy to crystallise and has been used in many investigations as a model system, also at XFELs^18^. This data deposition contains 9 different runs recorded using 4 different jet speeds. Each run has enough data to yield a structure in agreement with the known HEWL structure for all jet speeds. This data deposition contains both the raw and calibrated AGIPD data as well as the detector calibration constants used to calibrate the raw data. These data are suitable for algorithm development and testing for detector calibration, image classification and structure determination for use in future SFX experiments.

## Methods

### Sample preparation and delivery

Microcrystals of HEWL of size approximately 2 × 2 × 2 *μ*m were grown using an established protocol^18^ and transferred to a storage solution of 10% NaCl, 0.1 M sodium acetate buffer with pH 4.0. A 25% (v/v) suspension was prepared and filtered through stainless steel frits with pore sizes of 20 and 10 *μ*m before sample injection.

The filtered solution containing crystals was injected into the XFEL beam by gas dynamic virtual nozzles (GDVN) with helium as the focusing gas. The capillaries connecting the sample and gas reservoirs to the GDVN were each 2 m long and had inner and outer diameters of 100 and 360 *μ*m respectively. The GDVN was 3D printed using a customised computer-aided design based on *Design 6* by Knoška *et al*.^19^, The nozzle had a liquid orifice diameter of 75 *μ*m, a gas orifice diameter of 60 *μ*m and a distance between the liquid and gas orifices of 75 *μ*m. The production of the GDVN is described in detail by Knoška *et al*.^19^.

Datasets were recorded for 4 different jet velocities. The sample delivery parameters are described in Table 1.

**Table 1:** Description of sample delivery conditions and corresponding run number.

Run number	Duration (mins)	Flow rate (*μ*L/min)	He pressure (psi)	He flow rate (mg/min)	Jet velocity (m/s)	Frame count	Indexed count	Indexing rate (%)
79	10.1	30	450	34.0	50.8	2,129,547	47,129	2.21
80	12.1	30	450	34.0	50.8	2,564,884	20,156	0.79
84	10.0	80	400	26.0	37.4	2,116,224	20,479	0.97
85	10.0	80	400	26.0	37.4	2,116,224	30,401	1.44
95	10.0	60	450	34.0	44.0	2,113,760	31,697	1.5
96	10.0	60	450	34.0	44.0	2,113,760	40,239	1.9
98	10.0	60	300	14.0	31.2	2,115,520	40,802	1.93
99	10.2	60	300	14.0	31.2	2,154,241	74,437	3.46

### Experimental parameters

This experiment was performed at the SPB/SFX instrument^12^ at the European XFEL in March, 2020. Microcrystals of HEWL in random orientations were illuminated by 9.3 keV X-ray pulses focused to a full-width-at-half-maximum of approximately 3.2 *μ*m (horizontal) × 6.2 *μ*m (vertical) at the interaction point. The AGIPD was located 129 mm downstream of the interaction point and recorded 300 X-ray pulses per train with an intratrain repetition rate of 1.1 MHz. The average pulse energy upstream of the focusing optics was 1.6 mJ, the pulse resolved X-ray energy is also included in the data deposition.

**Fig. 1:**
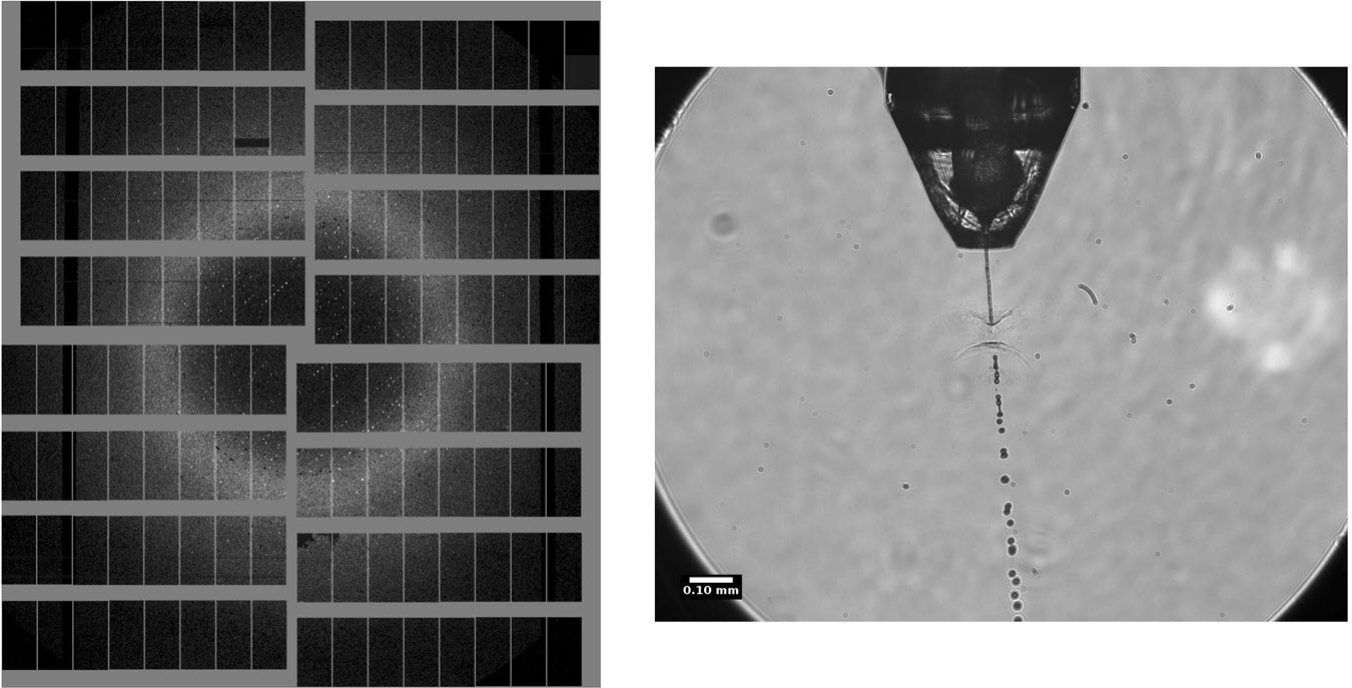
Example of single crystal diffraction data measured by AGIPD (left). Off-axis microscope for monitoring the overlap of the liquid jet and X-ray beam (right). The image was acquired with a single 800 nm wavelength, 65 fs duration laser pulse from the SASE1 pump-probe laser system, 110 ns after the first X-ray pulse in the train.

An off-axis microscope (Andor Zyla sCMOS with 10×  objective) having an effective pixel size of 1.3 *μ*m recorded the X-ray-liquid-jet interaction at 10 Hz and is included in the data deposition (see Data Records section). The liquid jet was illuminated by the 800 nm SASE1 femtosecond pump-probe laser^20^. The illumination laser was operated at 10 Hz with each pulse arriving at the interaction point 110 ns after the first X-ray pulse in each train. An example image is shown in Fig. 1. The jet velocity was determined by measuring the distance the exploded part of the jet travelled in a known time. Depending on the jet speed, this was either determined by the time between subsequent X-ray pulses in a train or by shifting the illuminating laser delay a known amount^21^. These measurements were taken between runs and are not part of the data set.

### Detector calibration

The AGIPD consists of 16 modules of *x* = 128 × *y* = 512 pixels each. The detector has three gain stages to cover the high dynamic range of one to several thousands photons per pixel. Each pixel has 352 analog memory cells (*mc*) which can store up to 352 images which consist of signal and gain information. The intensity measured in each AGIPD pixel and memory cell is described by two analog values, the analog signal and gain stage information^13^. To calibrate this raw signal, the relevant set of calibration constants is required. The calibration constants are derived using dedicated data sets. The set of constants required for calibrating the raw data are also included in the data deposition.

The list of calibration constants for each of the 16 AGIPD modules is provided in Table 2. The *gain* = 3 dimension indexes the high, medium or low gain stage. The *SlopesFF* array contains the relative high gain slope and intercept for first and second entries respectively and are generated from separate single photon flat field intensity measurements for identification of the single photon peak position. The constants in *SlopesPC* contain the *l* = 11 coefficients derived from the fit of the following functions to the data collected with the internal calibration source, the so-called pulsed capacitor data, used to scan high and medium intensity regions. First the linear region of the high gain stage is fit with the linear function:

1 $$y={c}_{0}x+{c}_{1},$$

where *c*_*l*_, for $l\in \mathrm{0...10}$ describe the data with index *l* in the *SlopesPC* constants. The high gain to medium gain transition and medium gain region is then fit with:

2 $$y={c}_{7}\,\exp \left(\frac{x+{c}_{5}}{{c}_{6}}\right)+{c}_{3}x+{c}_{4}.$$

**Table 2:** Calibration constants and corresponding file addresses and data dimensions used for calibrating the raw data from each of the 16 AGIPD modules at SPB/SFX.

Parameter	HDF5 key	Data dimensions
BadPixelsDark	/BadPixelsDark/0/data	(*x*, *y*, *mc*, gain)
BadPixelsFF	/BadPixelsFF/0/data	(*x*, *y*, *mc*)
BadPixelsPC	/BadPixelsPC/0/data	(*mc*, *x*, *y*)
Noise	/Noise/0/data	(*x*, *y*, *mc*, gain)
Offset	/Offset/0/data	(*x*, *y*, *mc*, gain)
SlopesFF	/SlopesFF/0/data	(*x*, *y*, *m* = 2)
SlopesPC	/SlopesPC/0/data	(*l* = 11, *mc*, *x*, *y*)
ThresholdsDark	/ThresholdsDark/0/data	(*x*, *y*,* mc*, *n* = 5)

The remaining parameters contain the residuals of the fit to the data. Parameter *c*_2_ describes the absolute relative deviation from linearity for the high gain region, *c*_8_ describes the absolute relative deviation from the linear part of the function in the medium gain region and *c*_9_ describes the threshold value for high and medium gain separation. The last parameter, *c*_10_, is unused in the current calibration implementation.

The *ThresholdsDark* array contains the gain state thresholds between high gain and medium gain, the threshold between medium gain and low gain and the gain values for high, medium and low gains for *n* = 0…4 respectively and are applied on a per pixel, per memory cell basis.

The calibration process consists of the following steps:

1. Gain stage identification
  To be able to identify the gain stage for each pixel and memory cell, so called gain thresholding has to be performed. For this, the analogue gain signal of each pixel and memory cell is evaluated against two thresholds values from *ThresholdsDark*.
2. Offset correction
  In this step, the appropriate gain stage offset from the Offset array is subtracted from the raw data.
  It was observed that the intensities for some pixels in offset corrected images (using the constants derived from dark data) gets negative values and the effect get stronger for the higher intensities. To partially mitigate the issue we decided to use an opaque mask (‘stripes’) which occlude a small area of each detector module. Using the information from this “shadowed” area, the additional ‘offset’ adjustment on per image basis should be performed. The “baselineshift” offset value is calculated for each module separately.
3. Gain correction

Depending on the gain stage, memory cell, x and y position, a gain correction value is multiplied with the result of the previous step.

In addition, for pixels identified to be in Medium Gain stage additional offset is added (i.e. intercept from linear fit for MG which can be found in *SlopesPC* array).

Further information on calibration of AGIPD data and the generation of calibration constants can be found in the EuXFEL Report by J. Sztuk-Dambietz^22^.

### Structure refinement

Each recorded run was processed independently using the CrystFEL software suite, version 0.9.1^23^. Each frame was processed using peakfinder8 for peak identification and subsequent peaks were indexed using MOSFLM. Conservative values were used for the Bragg peak finding in this case. It has recently been shown that with improved hit-finding parameters and algorithms the number of frames where crystal diffraction is detected is greatly increased^24^. The integrated intensities were merged and processed using XSCALE from the XDS package^25^. Resulting reflection files were then passed to phenix.phaser using the PHENIX package GUI^26^. Molecular replacement methods were used to borrow phases from a modified lysozyme model (PDB:1IEE) where side-chains with multiple conformations were simplified to that with the highest occupancy. FreeR flags were added to 5% of the data via phenix, prior to any model refinement steps. Default model refinement steps, such as simulated annealing, rigid body, reciprocal space, and real space refinement were performed to acceptable data quality. The resulting unit cell parameters are shown in Tables 3–5.

**Table 3:** Individual data quality statistics and figures of merit for Run 79, Run 80 (50.8 m/s) and Run 95, Run 96 (44.0 m/s).

Data Collection	Run 79	Run 80	Run 95	Run 96
space group	P 43 21 2	P 43 21 2	P 43 21 2	P 43 21 2
cell dimensions (Å)	79.73, 79.77, 38.60	79.73, 79.77, 38.60	79.73, 79.77, 38.60	79.78, 79.77, 38.61
cell dimensions (_°_)	90, 90, 90	90, 90, 90	90, 90, 90	90.02, 90.02, 90.03
Resolution	27.73 - 2.00 (2.07 - 2.00)	27.73 - 2.00 (2.07 - 2.00)	27.73 - 2.00 (2.07 - 2.00)	27.74 - 2.00 (2.05 - 2.00)
Rsplit	12.49 (111.33)	17.93 (141.74)	15.89 (173.28)	14.43 (175.84)
CC1/2 (%)	98.38 (50.57)	96.38 (31.59)	98.00 (27.67)	97.99 (23.48)
CC* (%)	99.59 (81.96)	99.07 (69.29)	99.49 (65.84)	99.49 (61.67)
SNR	6.75 (1.31)	4.61 (1.07)	5.46 (0.97)	6.18 (0.98)
Completeness	100 (100)	100 (100)	100 (100)	100 (100)
Multiplicity	421.3 (271.2)	174.1 (111.0)	246.6 (156.9)	342.8 (220.8)
**Refinement**				
No. reflections	16102	16102	16109	16190
Rwork/Rfree	0.1982/0.2279	0.1838/0.2092	0.1935/0.2327	0.1877/0.2318
Bond lengths (Å)	0.002	0.004	0.004	0.007
Bond angles (_°_)	0.49	0.63	0.58	0.79

**Table 4:** Individual data quality statistics and figures of merit for Run 84, Run 85 (37.4 m/s) and Run 98, Run 99 (31.2 m/s).

Data Collection	Run 84	Run 85	Run 98	Run 99
space group	P 43 21 2	P 43 21 2	P 43 21 2	P 43 21 2
cell dimensions (Å)	79.75, 79.75, 38.60	79.75, 79.75, 38.60	79.75, 79.75, 38.60	79.75, 79.75, 38.60
cell dimensions (_°_)	90, 90, 90	90, 90, 90	90, 90, 90	90, 90, 90
Resolution	27.73 - 2.00 (2.07 - 2.00)	26.20 - 2.00 (2.07 - 2.00)	27.73 - 2.00 (2.07 - 2.00)	27.73 - 2.00 (2.07 - 2.00)
Rsplit	21.44 (305.52)	16.60 (245.94)	15.79 (505.80)	11.00 (173.59)
CC1/2 (%)	96.47 (5.42)	97.95 (25.98)	98.01 (12.41)	99.01 (46.56)
CC* (%)	99.09 (32.07)	99.48 (64.22)	99.49 (46.98)	99.75 (79.71)
SNR	3.84 (0.55)	4.83 (0.71)	5.81 (0.46)	7.58 (0.85)
Completeness	100 (100)	100 (100)	100 (100)	100 (100)
Multiplicity	149.71 (95.09)	149.71 (95.09)	416.59 (263.80)	668.4 (431.5)
**Refinement**				
No. reflections	16109	16112	16079	16081
Rwork/Rfree	0.2005/0.2383	0.1874/0.2378	0.1973/0.2410	0.1950/0.2367
Bond lengths (Å)	0.002	0.004	0.004	0.003
Bond angles (_°_)	0.44	0.58	0.59	0.55

**Table 5:** Data quality statistics and figures of merit for runs combined by jet velocities.

Data Collection	Combined 31.2 m/s	Combined 37.4 m/s	Combined 44.0 m/s	Combined 50.8 m/s
space group	P 43 21 2	P 43 21 2	P 43 21 2	P 43 21 2
cell dimensions (Å)	79.75, 79.75, 38.60	79.75, 79.75, 38.60	79.75, 79.75, 38.60	79.73, 79.77, 38.60
cell dimensions (°)	90, 90, 90	90, 90, 90	90, 90, 90	90, 90, 90
Resolution	27.73 - 2.00 (2.07 - 2.00)	27.73 - 2.00 (2.07 - 2.00)	27.73 - 2.00(2.07 - 2.00)	27.73 - 2.00 (2.07 - 2.00)
Rsplit	9.43 (56.12)	12.45 (52.03)	14.21 (83.80)	12.79 (37.65)
CC1/2	99.1 (82.2)	98.9 (86.3)	98.5 (74.9)	97.9 (82.3)
CC*	99.7 (93.6)	99.5 (92.7)	99.5 (85.3)	99.5 (95.6)
SNR	7.44 (1.42)	6.09 (1.76)	4.52 (0.98)	6.44 (2.25)
Completeness	99.8 (100)	99.9 (100)	99.9 (100)	99.9 (100)
Multiplicity	549.81 (371.5)	269.3 (180.6)	160.4 (106.8)	301.8 (202.1)
**Refinement**				
No. reflections	15537	15554	15554	15548
Rwork/Rfree	0.1978 / 0.2346	0.1845 / 0.2296	0.2039 / 0.2232	0.1859 / 0.2300
Bond lengths (Å)	0.003	0.002	0.002	0.002
Bond angles (°)	0.53	0.44	0.41	0.44

## Data Records

The data deposited in the Coherent X-ray imaging Data Bank (CXIDB)^27^ contains approximately 19 million images in HDF5 format. The data set is divided up into runs which each contain about 10 minutes of data collection. The runs are further split across multiple HDF5 files. Raw data are located in the raw directory inside each run. Data are grouped in files according to detector and timestamp. Each AGIPD module is stored in a different file while other 10 Hz data are stored across other files. For example, the first 500 trains of data from AGPID module number 0 are stored in the file RAW-R0083-AGIPD00-S00000.h5. The calibrated data are then stored in CORR-R0083-AGIPD00-S00000.h5. The first 5000 trains of data in run 83 from the off-axis 10 Hz microscope are stored in data aggregator 3 file: RAW-R0083-DA03-S00000.h5. Data in files: CORR-RXXXX-AGIPD1MCTRLXX-SXXXXX.h5 contains detector specific configurations which are for beamline debugging purposes and not relevant to this data. Further information and description of the data can be found in the online European XFEL data analysis documentation^28^. The data can be found in ref. ^17^. A description of relevant data and process variables is given in Tables 6, 7.

**Table 6:** Relevant data sources and corresponding addresses within the deposited raw HDF5 data files.

HDF5 key (raw data)	Description & file
INSTRUMENT SPB_DET_AGIPD1M-1 DET 0CH0:xtdf image data	AGIPD raw intensity and gain bit for module 0. File: RAW-RXXXX-AGIPD00-SXXXXX.h5
INSTRUMENT SPB_EXP_ZYLA CAM 1:daqOutput data image pixels	off-axis microscope, monitoring the interaction region at 10 Hz File:: RAW-RXXXX-DA03-SXXXXX.h5
INSTRUMENT SPB_XTD9_XGM XGM DOOCS:output data intensitySa1TD	X-ray pulse energy measured upstream of the instrument (*μ*J). File: RAW-RXXXX-DA01-SXXXX.h5
INSTRUMENT SA1_XTD2_XGM XGM DOOCS:output data intensitySa1TD	X-ray pulse energy measured downstream of the SASE1 undulator (*μ*J). File: RAW-RXXXX-DA01-SXXXXX.h5
CONTROL SPB_IRU_AGIPD1M MOTOR Z_STEPPER actualPosition value	AGIPD positioner stage readback value (mm). File: RAW-RXXXX-DA03-SXXXXX.h5
CONTROL ACC_SYS_DOOCS CTRL BEAMCONDITIONS kParameter value	SASE1 undulator k-parameter (). File: RAW-RXXXX-DA01-SXXXXX.h5

**Table 7:** Relevant data sources and corresponding addresses within the deposited calibrated HDF5 data files.

HDF5 key (calibrated data)	Description
INSTRUMENT SPB_DET_AGIPD1M-1 DET 0CH0:xtdf image data	AGIPD raw intensity and gain bit for module 0
INSTRUMENT SPB_DET_AGIPD1M-1 DET 0CH0:xtdf image gain	AGIPD gain state for module 0
INSTRUMENT SPB_DET_AGIPD1M-1 DET 0CH0:xtdf image mask	AGIPD pixel mask for module 0

## Technical Validation

The calibrated diffraction data were analysed using the CrystFEL software suite^23^. The resulting unit cell showed excellent agreement with the well known HEWL unit cell, the unit cell parameters for each run are described in Tables 3–5. The unit cell parameters for run 97 are not shown but are almost identical to those found in run 96.

## Data Availability

Data was analysed with CrystFEL 0.9.1. The CrystFEL 0.9.1 software suite is a free open source software available under the GNU Public License version 3 and can be downloaded from http://www.desy.de/twhite/crystfel/. The AGIPD data was calibrated using the EuXFEL calibration pipeline, release 3.0.0-beta^29^. The raw data and calibration constants are also available for development of calibration algorithms.
